# The Neurophysiology of Caffeine as a Central Nervous System Stimulant and the Resultant Effects on Cognitive Function

**DOI:** 10.7759/cureus.15032

**Published:** 2021-05-14

**Authors:** Brian Fiani, Lawrence Zhu, Brian L Musch, Sean Briceno, Ross Andel, Nasreen Sadeq, Ali Z Ansari

**Affiliations:** 1 Neurosurgery, Desert Regional Medical Center, Palm Springs, USA; 2 College of Osteopathic Medicine, New York Institute of Technology, Glen Head, USA; 3 College of Osteopathic Medicine, William Carey University, Hattiesburg, USA; 4 College of Osteopathic Medicine, Lake Erie College of Osteopathic Medicine, Erie, USA; 5 School of Aging Studies, University of South Florida, Tampa, USA; 6 College of Behavioral and Community Sciences, University of South Florida, Tampa, USA

**Keywords:** methylxanthine, cognitive performance, fluid intelligence, reaction time, mental alertness, mood and anxiety

## Abstract

Caffeine is one of the world’s most consumed drugs. According to the Washington Post (2015), two billion cups of coffee are consumed per day worldwide. Caffeine is classified as a central nervous system (CNS) stimulant and an organic molecule called methylxanthine. Caffeine has three notable mechanisms of action on the CNS that produce a psychostimulant effect. These effects are responsible for the effect that caffeine has on cognitive function. The effects of caffeine consumption on cognitive function have been demonstrated across several studies involving humans and animals. With the immense number of people consuming caffeine around the world, it is of vital importance to study the effects that this drug has on people’s cognitive function. This literature review provides useful insights on this question through the analysis of caffeine’s effects on cognitive function, along with information on caffeine’s three modes of action. The findings of recent studies show mixed results regarding the effects of caffeine on mood, attention, processing speed, and memory. Current research suggests that if caffeine does have an effect on mood, the most significant changes may be anxiety. Studies did not support caffeine as having any significant effect on attention, but that it did play a role in enhancing processing speed. The majority of the studies reviewed suggest caffeine as having a significant positive effect on both short and long-term memory in adults and the elderly. Current findings warrant continued research on the association of caffeine and the resultant effects on cognitive function.

## Introduction and background

Caffeine is one of the world’s most consumed drugs, and is consumed in various forms such as coffee, energy drinks, soda, or chocolate. According to the Washington Post (2015), two billion cups of coffee are consumed per day worldwide [1]. Caffeine consumption affects the cognitive function of its consumers in a variety of different ways. Caffeine has been shown in studies to help enable the learning and memory of tasks in which information is passively presented [2,3]. In addition, caffeine has been shown to improve performance in tasks upon which the working memory is dependent on to an extent [4]. Caffeine has also been found to lower the consumer’s anxiety levels and improve their hedonic tone when consumed in small doses [5,6]. With so many people around the world consuming caffeine daily, it is of vital importance to study the effect that this drug has on people’s cognitive function. We sought to review the mechanism of action that caffeine has on the brain, as well as look at recently reported studies investigating the effects of caffeine on users, specifically with outcomes of mood, memory, processing speed, and attentiveness.

## Review

Mechanism of action

Caffeine is classified as a central nervous system (CNS) stimulant and an organic molecule called methylxanthine. Caffeine has three notable mechanisms of action on the CNS that produces a psychostimulant effect. These effects are responsible for the influence caffeine has on cognitive function. The effects of caffeine consumption on cognitive function have been demonstrated across several studies involving humans and animals. For example, Angelucci et al. tested the effects of caffeine on memory in rats using the Morris water maze task [7]. The rats in this study were either given caffeine immediately after training, 30 minutes before training, or 30 minutes before they were to be tested in the maze [7]. The study concluded that caffeine consumption was linked to greater memory retention in rats; however, the consumption of caffeine was not related to memory acquisition [7]. The first mechanism of action for caffeine involves methylxanthine stimulating antagonism at the adenosine receptor level. Methylxanthine compounds, such as caffeine, can act as a competitive antagonist against the depressant effects of adenosine [8]. In the brain, adenosine and adenosine receptors regulate the release of neurotransmitters and play an important role in the regulation of sleep, arousal, cognition, memory, and learning [9]. Caffeine binds to adenosine receptors, which in turn block the binding of adenosine to its receptor. The blockage of adenosine receptors indirectly affects the release of neurotransmitters such as norepinephrine, dopamine, acetylcholine, serotonin, glutamate, and gamma-aminobutyric acid (GABA). An influx in these neurotransmitters alters mood, memory, alertness, and cognitive function [4].

A second mechanism of action for caffeine can be described as the effect of methylxanthine on the mobilization of intercellular calcium. Caffeine which is a methylxanthine compound promotes the movement of calcium through the sarcoplasmic reticulum and the plasma membrane. This calcium is then released via synaptic transmission into the peripheral and central nervous systems that are dependent on a controlled release of neurotransmitters. These neurotransmitters, in turn, are dependent upon the calcium influx that travels into the nerve endings. At low concentrations of methylxanthine, the uptake and release of calcium facilitated through the endoplasmic reticulum increase. However, at higher concentrations of methylxanthine, the uptake of calcium by the endoplasmic reticulum is inhibited [8]. The effects of caffeine that are felt through this mechanism are not due to suppression of adenosine, GABA, or noradrenaline. In the body there are three distinct intracellular pools of calcium that have been defined through the use of their turnover number and their specific mechanisms of action [8]. In this mechanism, pools two and three are sensitive to the release of calcium stimulated by caffeine intake at low doses. This mode of action for caffeine is not as likely as methylxanthine’s antagonistic effect of adenosine due to the higher concentration of caffeine needed for this mechanism of action to be a viable option [8].

The third mechanism of action for caffeine involves the ability of methylxanthine to inhibit phosphodiesterases. Methylxanthine prevents cAMP from being enzymatically broken down. Methylxanthine does this by inhibiting the cyclic nucleotide phosphodiesterase, which stimulates the accumulation of cAMP. The accumulation of cAMP then stimulates the release of hormones such as dopamine, epinephrine, and noepinephrine. An influx in these neurotransmitters alters mood, memory, alertness, and cognitive function [4]. However, this mode of action is not as likely as methylxanthine’s antagonistic effect of adenosine because the concentration of methylxanthine needed for caffeine to utilize this mechanism would be considered toxic to humans [8].

Trials and outcomes

To evaluate the effects of caffeine consumption on mood and cognitive performance, national databases were used to identify recent studies that reported subjective and objective outcomes of caffeine consumption across populations. Specifically, studies were identified with data on the impact of caffeine consumption on mood, attention, processing speed, and memory. A summary of studies included in this review is presented in Table 1.

**Table 1:** Studies investigating the effects of caffeine on cognitive function.

Author, year	Population	Caffeine source	Outcomes of interest	Outcome findings
Araújo et al., 2015 [10]	n = 14,563 35-74 years old	<1 cup to >3 cups per day	Attention, processing speed, memory	No effect on attention or processing speed. Improved memory in the elderly (65-74 years old)
Benson et al., 2019 [11]	n = 24 18-40 years old	Red Bull (80 mg)	Memory	Improved working memory reaction time
Cornelis et al., 2020 [12]	n = 434,900 37-73 years old	Caffeine (any amount) within the last hour	Processing speed, memory	Improved reaction time. Impaired memory
Franceschini et al., 2020 [13]	n = 53	Caffeine (200 mg)	Mood, memory	No effect on mood and memory
Garcia et al., 2017 [14]	n = 80 (medical students)	Drink A (174.5 mg) Drink B (147.2 mg) Drink C (155 mg)	Mood, attention	No effect on mood and attention
Marczinski et al., 2014 [15]	n = 14 18-29 years old	5 Hour Energy (200 mg)	Mood	Positive effects on vigor-activity and tension-anxiety. No effect on other mood states
Repantis et al., 2021 [16]	n = 48 21-36 years old	Caffeine (200 mg)	Attention, processing speed	Positive effects on mood and processing speed
Sherman et al., 2016 [2]	n = 83 18-21 years old	Decaffeinated coffee (7-10 mg)/Coffee (180 mg)	Memory	Increased memory during non-optimal time of day – early morning
Smith, 2013 [3]	n = 128 18-65 years old	Decaffeinated coffee/Coffee (65 mg)	Processing speed, memory	Increased processing speed. No effect on memory
Thomas et al., 2019 [17]	n = 9 19-23 years old	Ai Reload (AiR) (130 mg)	Attention, processing speed, memory	No effect on attention and memory. Increased processing speed
Wesnes et al., 2017 [18]	n = 25 19-33 years old	Red Bull (80 mg)	Mood, attention, memory	Increase in “alert” and “jittery” and a decrease in “tired.” No effect on attention. Increased information retrieval speed (component of working memory)
Zabelina et al., 2020 [19]	n = 88 18-35 years old	Caffeine capsule (200 mg)	Mood, memory	Decrease in sadness, no effect on other mood states. No effect on working memory
Zhang et al., 2020 [20]	n = 11,875 9–10 years old	Coffee, tea, soda, espresso, and energy drinks. Daily average caffeine intake of 13.00 + 43.73 mg	Memory, processing speed	Negative effect on working memory and episodic memory. Negative effects on processing speed

Mood

Dose-dependent effects of caffeine and increased energetic arousal have been studied extensively; however, the relationship between caffeine use and individual mood states has not been thoroughly evaluated. Recently, several studies have investigated the effects of caffeine on mood states such as anxiety, vigor, alertness, anger, and sadness.

In a randomized, placebo-controlled study, Marczinski et al. examined the effect of caffeine (5 Hour Energy Drink, 200 mg caffeine) on six different moods [15]. This included tension-anxiety, depression-dejection, anger-hostility, vigor-activity, fatigue-inertia, and confusion-bewilderment. A total of 14 individuals (ages 18 to 29) were assessed with the Profile of Mood States (POMS) Brief form [21], which is known to be sensitive to the acute administration of caffeine on mood. No significant differences were found in the mean POMS rating scores for the six mood states compared to the baseline following caffeine administration (p > 0.24). Two of the six moods, namely, vigor-activity and tension-anxiety, had a significant main effect of dose and time and dose-time interaction, respectively. Analysis of variance (ANOVA) for the POMS vigor-activity rating revealed a significant main effect of caffeine dosage and a significant main effect of time post-administration (p = 0.002). Post-hoc least significant difference (LSD) tests revealed that vigor-activity scores were significantly higher post-caffeine administration compared to both the placebo and no drink groups (p < 0.043), with no significant difference between the placebo and no drink group (p = 0.10). Vigor-activity rating declined with time, suggesting a significant increase in subjective vigor-activity post-caffeine consumption in a dose-, but not time-, dependent manner. ANOVA for tension-anxiety ratings on the POMS suggested a significant dose-time interaction (p = 0.044). Interestingly, Post-hoc LSD analysis on tension-anxiety ratings revealed that ratings were significantly higher at 40 minutes post-caffeine use than any other time point (40 to 340 minutes), especially in comparison to no drink conditions (p = 0.028), suggesting that caffeine consumption might have a brief alteration in mood states (i.e., anxiety) which are short-lived (<1 hour).

Garcia et al. utilized a different method to evaluate caffeine and mood using 80 medical students (50 men and 30 women) recruited into a randomized, placebo-controlled study [14]. The State-Trait Anxiety Inventory (STAI), a 20-item questionnaire, was administered prior to cognitive testing to evaluate psychological stress in response to caffeine. Each question is evaluated using a set ordinal scale (not at all = 1, very much = 4). The total score correlates with a certain anxiety level. Results showed a significant decrease in anxiety for participants who received Drink C (155 mg caffeine) compared to groups that received Drink A (149.5 mg caffeine) and B (147.2 mg caffeine). However, the percentage change was not different among Drink A, Drink B, and Drink C compared with the control. Interestingly, salivary cortisol levels were also measured as an objective evaluation of biological stress. Results showed a significant increase in Drink B; however, similar to the STAI scores, there was no significant percentage increase in cortisol levels among the three groups compared to the control. Thus, findings on the effect of caffeine on biological stress and anxiety were inconclusive.

A similar, double-blind study by Franceschini et al. assessed the effect of caffeine on cognitive performance and looked at anxiety, specifically [13]. Anxiety was evaluated at 30 minutes post-caffeine use. The authors also administered the STAI to examine mood in 53 individuals (low-to-normal habitual caffeine consumers). Self-evaluations were scored. Similar to the findings of Garcia et al., results showed no significant difference between caffeine and placebo consumption and anxiety level (p = 0.136) [13].

A more recent study by Zabelina et al. evaluated the effects of caffeine on working memory and mood [19]. A total of 88 participants (60 females, 28 males; mean age = 21.6 years) were recruited into a randomized, double-blind, placebo-controlled study. Participants were asked to indicate on a sliding scale, from 0 (not at all) to 100 (very), to what extent they were feeling happy, sad, excited, anxious, bored, and focused before and after caffeine intake. t-tests performed on individual mood states indicated that caffeinated subjects reported a decrease in sadness after caffeine consumption, the control group reported an increase in sadness post-consumption (p = 0.027). However, no significant differences were reported in the remaining mood states (p > 0.336), including that of anxiety. Subjective anxiety levels in the caffeinated group exhibited no significant change compared to control (p = 0.633). The results were consistent with the previous two studies showing no significant alterations in mood with caffeine use. However, the significant decrease in sadness observed in this study may be because caffeine is frequently associated with increased energetic arousal. A randomized, double-blind, placebo-controlled, three-way, cross-over trial by Wesnes et al. utilized the Caffeine Research visual analogue scale (VAS) to evaluate the potential mood changes associated with drinking Red Bull energy drink (80 mg caffeine) in 24 adults (ages 19-33 years) [6,18,22]. Mixed-model repeated-measures (MMRM) and ANOVA tests were conducted on the changes from baseline. The Caffeine Research VAS displayed significant main effects in “alert” (p = 0.0005), “tired” (p = 0.0356), and “jittery” (p = 0.0044). Individuals who consumed Red Bull showed significant increases over baseline in “alert” and “jittery” and a significant decrease under baseline for “tired.” This pattern suggested that the consumption of energy drinks, such as Red Bull, may increase energetic arousal and may be correlated with decreased sadness shown in the study performed by Zabelina et al. [19].

Attention and processing speed

Attentiveness or attention pertains to the ability of an individual to focus on information relevant to an assigned task while suppressing other less relevant information available. Processing speed pertains to the speed at which an individual is able to detect and respond to rapid changes in the environment. Of the five studies under review that examined the relationship between caffeine and attention, four studies showed no significant association between attention and caffeine use, while a more recent study showed a positive correlation. Of these, four of the studies also examined processing speed in which two showed a positive correlation while the other two showed no significant change in processing speed in relation to caffeine consumption.

In a cross-sectional study, Araujo et al. studied the association between coffee consumption within the last 12 months and cognitive function [10]. The data were part of a longitudinal study, The Longitudinal Study of Adult Health (ELSA-Brasil, 2008), that examined the development and progression of chronic illnesses among 15,105 civil servants in Brazil [23]. A total of 14,563 participants were separated into two age groups: 35-64 and 65-74 years old. Participants who underwent cognitive testing also reported having consumed caffeine for the last 12 months (<1 cup to >3 cups per day). Trail-making tests were administered to examine executive functioning relating to attention and psychomotor speed. Prior to adjusting for age, sex, and education, data showed a 3% improvement in mean time taken to complete the trail-making test (p = 0.025). Despite results from the tests showing statistical significance, there is not enough information to indicate that coffee consumption altered mean performance in this age group (35-64 years old). For individuals in the 65-74-year-old age group, no significant findings were observed in the trail-making test results. There are no indications for improvements in attention or processing speed related to caffeine consumption. A dose-dependent relationship was also not observed.

The study that evaluated caffeine’s effect on mood by Wesnes et al. also looked at cognitive performance in 24 participants (ages 19-33 years) after consuming Red Bull (80 mg caffeine) [18]. The CogTrack system, a set of nine cognitive tests, was administered 30 minutes post-drink on the computer. Changes from baseline scores for each test were used to evaluate attentional intensity, sustained attention, and speed of retrieval indices. Overall, Red Bull did not produce a significant main effect on attentional intensity and sustained attention indices. Red Bull consumption showed a significant increase in the speed of retrieval index (t = 4.02, p = 0.0002), which was not seen in the placebo (t = 0.35, p = 0.728) or Sugar-Free Red Bull (80 mg caffeine) (t = 1.46, p = 0.1482), demonstrating potential positive effects of caffeine (Red Bull) on information retrieval speed, but not on attention.

Garcia et al. utilized the N-Back Task to assess attention and determine possible cognitive enhancements after caffeine ingestion [14,24]. The N-Back Task is a continuous performance task that is commonly used as an assessment in psychology and cognitive neuroscience to measure a part of working and working memory capacity. Results (percentage scores) were used to interpret attentiveness. Percentage change in N-Back Task results showed an increase after consuming Drink A (149.5 mg caffeine) compared to control, but no significant changes were observed in any of the individual groups following caffeine consumption.

With the popular demand to find ways to improve performance, both physically and mentally, energy drinks have become a popular go-to for many traditional athletes and e-sports athletes to improve attention and reaction time. Therefore, Thomas et al. conducted a study among nine elite League of Legends (LoL) e-sport players belonging to the same professional team [17]. In this randomized, double-blind, placebo-controlled, cross-over study, players were evaluated for cognitive changes associated with energy drink consumption, specifically Ai Reload (AiR) (130 mg caffeine), before competing in three LoL matches. Three cognitive assessments were administered: Eriksen Flanker Test (measures attentional ability), Go/No-go Visual Reaction Time Test, and Working Memory Test (N-Back Task previously described). Improvements were seen in mean reaction time from pre-game (668.9 ± 216.3 ms) to post game three (497.5 ± 105.1 m; p = 0.004); however, no other significant differences across treatments or times were identified. Ultimately, AiR did not improve performance parameters. Interestingly, all participants were found to habitually consume AiR prior to gaming, suggesting potential habituation to caffeine prior to the study. The small sample size in this unique population is a limiting factor. Larger sample sizes could provide statistically and clinically significant data in this unique group of athletes.

In contrast to the previous four studies, a recent study by Repantis et al. suggested improvements in cognitive parameters with caffeine use [16]. In a randomized, double-blind, placebo-controlled study, cognitive performance in 48 male participants (21-36 years old) was assessed after taking one of three stimulants (caffeine, methylphenidate (MHP), modafinil (MOD)). Attention was assessed via the Psychomotor Vigilance Test, a reaction time task established to measure speed of response to visual stimuli [25]. Response times were used to score participants’ attention and psychomotor speed. Compared to control, caffeine demonstrated a positive effect on sustained attention and significant shorter reaction times (p = 0.002), while MHP and MOD showed no significant improvements in either parameter. Results suggested that caffeine is unique in its ability to significantly affect cognitive performance in healthy individuals who do not require medication (MHP or MOD).

Memory

Another cognitive parameter of interest is memory, commonly separated into short-term (working) memory and long-term memory. Working memory briefly stores information while other cognitive processes are performed. For this reason, only limited amounts of information can be stored in short-term memory allowing us to manipulate information to execute complex, multi-step tasks. Long-term memory contains vast amounts of information that is stored for significantly longer periods of time compared to short-term memory. Some of this information can even be stored for a lifetime.

The majority of the studies reviewed have shown positive associations between caffeine and memory. A study performed by Smith recruited 128 individuals (1:1 male:female ratio; age 18-65 years) for a double-blind study that investigated the impact of caffeine consumption when performing working memory tasks and simple reaction time tasks [3]. ANOVA analysis demonstrated that although there were no significant main effects of caffeine on working memory, participants who received caffeinated coffee had significantly faster simple reaction times (322 m; p = 0.013) than those who received decaffeinated coffee (345 m). This suggested that a low dose of caffeine (65 mg) reduced simple reaction time and increased the coding of new information.

Previously discussed in attention and psychomotor speed, Araujo et al. also examined the association between chronic coffee consumption and memory [10]. Recall, recognition, semantic, and phonemic fluency tests were used to assess short-term and long-term memory. For Individuals aged 65-74, drinking two to three cups per day was associated with a 4% increase (p = 0.026) in the mean number of words remembered. Drinking more than three cups in this age group also increased the mean number of words pronounced in the semantic verbal fluency test by approximately 1.23 words (p = 0.016). No significant dose-dependent response relationship was noted, suggesting that caffeine was associated with improvements in memory in the elderly, but not in a dose-dependent manner.

Sherman et al., studied the effects of caffeine in undergraduate students from the University of Arizona (ages 18-21 years) [2]. Participants were asked to complete implicit and explicit memory tasks in the morning (6-8 a.m., non-optimal time of day) and in the afternoon (2-4 p.m., optimal time of day). Individuals who preferred mornings were excluded from the study via the Morningness-Eveningness Questionnaire [26]. A total of 23 studies performed in the morning showed that participants in the caffeinated group performed significantly better than the decaffeinated group on explicit memory tasks (p < 0.05). However, no significant differences were observed for implicit memory. In contrast to the morning session, caffeine did not influence implicit or explicit memory performance in the afternoon session. This suggested that caffeine is associated with enhancements in explicit memory in young adults during suboptimal conditions, such as early morning. Sherman et al. also analyzed the possibility of performance being influenced by the participants’ perception of caffeine being beneficial but found that perception alone did not influence explicit performance.

In the study performed by Wesnes et al., the consumption of Red Bull did not demonstrate a greater decline over the post-drink period in the memory capacity index [18]. However, it demonstrated a significant increase in the speed of retrieval index (p = 0.0002), as previously mentioned. These findings demonstrate the potential positive effects of caffeinated beverages (Red Bull) on memory performance and information retrieval speed.

A study performed by Benson et al. also evaluated the impact of Red Bull on cognitive performance [11]. A total of 24 individuals participated in a factorial, double-blind, placebo-controlled, crossover study to evaluate the impacts of alcohol and Red Bull on cognitive performance. A computerized test battery was used to evaluate the effects of caffeine on working memory. At 90-minute post-Red Bull consumption, working memory reaction time was significantly faster compared to placebo (p = 0.040).

Although many studies have shown positive associations between caffeine consumption and memory, two most recent studies have shown data demonstrating a negative association. Zhang et al., performed a population-based study which analyzed data from an ongoing Adolescent Brain Cognitive Development (ABCD) study to assess the effects of caffeine on cognitive function in children [20]. A total of 11,875 participants, aged 9-10, consisting of a similar proportion of males and females living in the United States were recruited [27]. Of the 11,875 participants, six did not report any information on caffeine use, 144 did not complete a cognitive battery test, and seven did not have data from either caffeine use or cognitive testing. Five different caffeinated beverages, such as coffee, tea, soda, espresso, and energy drinks, were included in the study with a daily average caffeine intake of 13.00 ± 43.73 mg/day (soda being the greatest contributor). According to the USDA National Nutrient Database, this average equates to approximately one-eighth of a can (12 fl oz) of AMP energy drink or two packs of M&M’s Milk Chocolate candy. In contrast to popular belief, Zhang et al. suggested that caffeine consumption had a negative correlation with working memory, episodic memory, and processing speed (p < 0.01), suggesting that children who chronically consumed caffeine exhibited a worsening in overall cognitive performance [20]. This may indicate that habitual caffeine consumption could have detrimental effects on neurologic development in children, especially during critical neurological developmental periods. With the consumption of soda, chocolate, and caffeinated beverages on the rise in the United States, this can become a growing concern. Negative correlations were also seen in a study by Cornelis et al. who investigated the impact of recent caffeine use (within the hour) on cognitive performance [12]. Interestingly, this study accounted for lifestyle and genetic factors that may impact caffeine metabolism which have not been studied extensively in the past. The study consisted of 434,900 UK Biobank participants (aged 37-73 years) recruited from 2006-2010, who provided biological samples and completed questionnaires regarding sociodemographic factors, medical history, lifestyle, and diet. Participants completed at least one of four self-administered cognitive function tests assessing prospective memory (PM), pairs matching (Pairs), fluid intelligence (FI), and reaction time (RT). Multivariable regression analyses were used to examine the association between recent caffeine consumption and cognition test scores. Results showed that recent caffeine use was associated with lower performance on PM, Pairs, and FI, which suggested potential impairments in memory and reasoning, while RT was not affected. Lifestyle factors such as smoking or habitual caffeine intake showed no significant or consistent effect modification on cognition.

Two additional studies reviewed showed that caffeine consumption did not always have an identifiable association with memory performance. In a double-blind, repeated-measures design study, Franceschini et al. evaluated the influence of caffeine consumption on lexical long-term and short-term memory in 53 volunteers [13]. To test long-term memory, participants had to take a multiple-choice questionnaire about information contained in a brief text they were asked to read. Results showed that performance was not modified by caffeine intake. Phonological short-term memory was assessed via participants listening to a series of pseudo word trigrams, a group of three consecutive written units such as letters, syllables, or words. They were then asked to repeat each trigram in the correct sequence and their performance was scored. Again, phonological short-term memory showed no influence by caffeine use. These results suggested that low-to-moderate caffeine consumption on a chronic basis was not associated with improvements in long-term or short-term phonological memory. Zabelina et al. evaluated the effects of caffeine on convergent and divergent thinking and working memory [19]. A total of 88 participants (60 females, 28 males) were asked to participate in the Keep Track task to assess working memory [28]. The task required volunteers to remember the last word to appear on the screen from two to five different categories (animals, colors, countries, distances, metals, and relatives). Recall accuracy was measured. t-tests suggested that there was no appreciable difference in working memory between subjects who consumed caffeine capsules and the control (p = 0.679).

## Conclusions

Caffeine consumption continues to be on the rise. Coffee, tea, energy drinks, and chocolate are more popular than ever in all age groups. Recent studies continue to show mixed results regarding the effects of caffeine on mood, attention, processing speed, and memory. Current research seems to suggest that if caffeine does have an effect on mood, the most significant changes can be seen in anxiety. Studies do not support caffeine as having any significant effect on attention, but it does seem to play a role in enhancing processing speed. The majority of the studies reviewed focused on the effects of caffeine on memory. Most of the data suggest caffeine having significant positive effects on both short- and long-term memory in the adult and elderly populations, but not in children. This raises concerns for the potential detrimental effects of caffeine in developing children and could be an area of research that would provide more clinical significance for the pediatric population. Many of the studies reviewed used energy drinks as their source of caffeine possibly due to the current popularity of energy drinks. This also raises the question regarding whether other compounds in these drinks have a synergistic effect or pose a potential harm to an individual’s cognitive and physical health. Further research with larger sample sizes will be needed to determine its significance.

Moreover, subjective outcomes of caffeine consumption on mood varied greatly. Future studies investigating the potential correlations between mood and caffeine should consider the impact of factors such as socioeconomic status, mental health, occupation, gender, age, and other lifestyle factors that may impact habitual caffeine use and underlying mood states. Additional investigation is also needed to determine the relationship between caffeine consumption, arousal state, and mood, and whether these effects are dose-dependent or independent of dose.
